# Evidence for temporal-coherence-based segregation of complex auditory scenes in the newborn human brain

**DOI:** 10.3389/fnhum.2026.1719515

**Published:** 2026-04-01

**Authors:** Silvia Polver, Petra Kovács, Gábor P. Háden, István Sziller, István Winkler, Brigitta Tóth

**Affiliations:** 1 HUN-REN Institute of Cognitive Neuroscience and Psychology, Research Centre for Natural Sciences, Budapest, Hungary; 2 Laboratoire des Systèmes Perceptifs, UMR CNRS 8248, Ecole Normale Supérieure, PSL University, Paris, France; 3 Department of Developmental Psychology and Socialisation, University of Padua, Padua, Italy; 4 Department of Cognitive Science, Faculty of Natural Sciences, Budapest University of Technology and Economics, Budapest, Hungary; 5 Acoustics Research Institute, Austrian Academy of Sciences, Vienna, Austria; 6 Faculty of Psychology, University of Vienna, Vienna, Austria; 7 Department of Telecommunications and Media Informatics, Faculty of Electrical Engineering and Informatics, Budapest University of Technology and Economics, Budapest, Hungary; 8 Division of Obstetrics and Gynaecology, DBC, Szent Imre University Teaching Hospital, Budapest, Hungary

**Keywords:** auditory stream segregation, event-related potentials, newborn infants, object-related negativity, temporal coherence

## Abstract

Detecting a target sound within a mixture of sounds (referred to as auditory stream segregation) is crucial for perception in natural environments, a skill humans and animals excel at. This study investigates the role of temporal coherence in auditory stream segregation in human newborns using high-density EEG recordings. Sleeping newborns were exposed to temporally coherent auditory tone sequences embedded in random background tones, and their event-related responses were analyzed. The results indicate that newborns can segregate auditory streams based on temporal coherence, suggesting that this stream segregation is driven by automatic mechanisms from birth, as evidenced by brain responses resembling the object-related negativity (ORN) event-related potential (ERP) component. However, discriminating among different signal-to-noise ratios requires further fine-tuning, as evidenced by delayed latencies in neonates compared to adults. These findings indicate that temporal coherence aids in detecting and orienting toward salient stimuli, thereby laying the foundation for the development of abilities such as selective attention and speech perception.

## 1 Introduction

In everyday life, we are often surrounded by multiple sounds playing at once, competing for our attention, such as conversations in crowded spaces, traffic noise, and background music. Separating sounds from this mixture (auditory stream segregation) is crucial for auditory perception in natural environments, a skill at which both humans and animals excel (Bregman, 1994). This process enables us to navigate and interpret complex auditory environments efficiently and is crucial for effective communication, speech comprehension, and sustained and selective attention. There are two primary forms of auditory stream segregation: concurrent or spectral and sequential stream segregation (Bregman, 1994). The former relies on spectral cues from the set of concurrently presented sounds, such as harmonic relationships, without reference to previous sounds. The latter provides cues extracted from the relationship between successive sounds, such as their similarity. There is, however, a cue, which is based on integrating concurrent and sequential stimulus features: temporal coherence, i.e., the dynamic co-modulation of sounds, which helps the brain to bind concurrent sounds together into perceptual streams, and separating them from sounds that follow different patterns of temporal modulation (Shamma et al., 2011; Teki et al., 2013). The present study tested neonatal auditory stream segregation based on temporal coherence, which requires integrating concurrently presented frequency components over time to form a coherent auditory object.

Much research has been conducted to understand the neural mechanisms and cognitive processes underlying auditory stream segregation (for recent reviews, see Hermes, 2023; Winkler and Denham, 2024). However, less is known about newborn infants’ innate capabilities. The present study aims to investigate the innate aspects of auditory stream segregation with respect to temporal coherence, focusing on the developmental emergence and neural mechanisms underlying auditory scene analysis.

### 1.1 The temporal coherence theory of auditory stream segregation

Temporal coherence refers to the joint modulation of the amplitudes of concurrent spectral elements of the incoming sound over time. It represents a general feature of our physical environment: all parts of a sound originating from the same source usually fluctuate together in time (Lu et al., 2017). This principle was first observed by the Gestalt school of psychology (termed common fate; Köhler, 1947) and is an essential factor in auditory stream segregation, probably because, unlike their visual counterpart, auditory scenes do not include static elements, which can be revisited for extracting further information (Boncz et al., 2024; O'Sullivan et al., 2015; Shamma et al., 2011; Teki et al., 2013; Tóth et al., 2016). The brain of adult listeners detects this coherence (O'Sullivan et al., 2015) and uses it to group concurrent sounds with similar temporal modulation, while separating them from concurrent sounds with different temporal modulation patterns. For example, temporal coherence helps us separate a speech stream from background noise by detecting consistent timing patterns in speech signals (Shamma et al., 2013).

**Figure 1:**
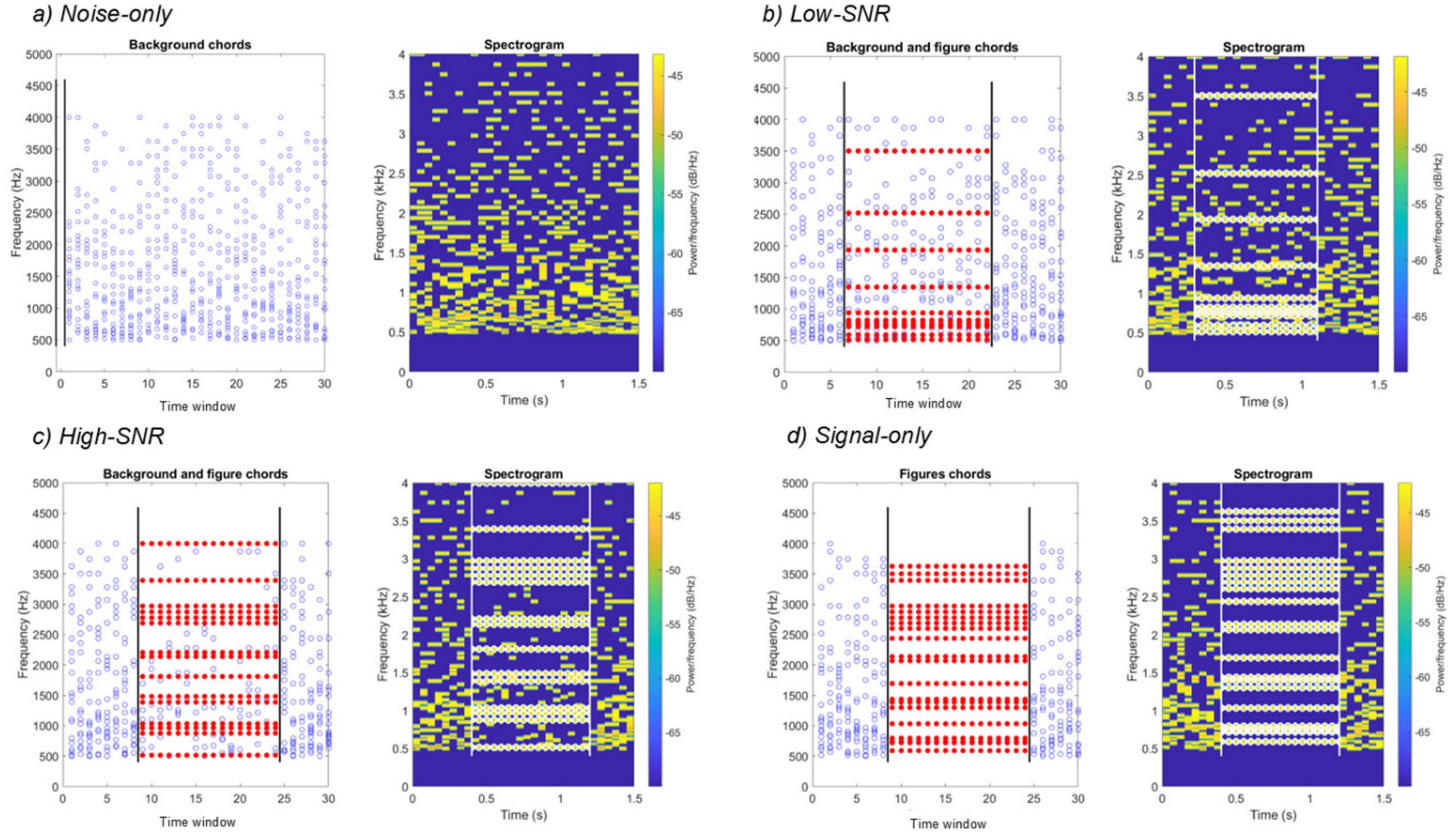
Spectrograms of the four experimental conditions. Blue circles (left panels) and yellow rectangles (right panels) represent tones. The *x*-axis represents time, and the *y*-axis represents frequency. The signal’s onset and offset are marked by vertical lines in **(b–d)**, and the tones in the signal are highlighted with red (left panels) or a white X (right panels). In the noise-only condition **(a)**, there is no signal, only background tones.

Several results support the role of temporal coherence in listening under noisy conditions, as demonstrated by psychoacoustic and neuroimaging studies using *stochastic figure-ground* (SFG) stimuli: repeating tonal patterns embedded within clouds of randomly varying pure tones (Boncz et al., 2024; O'Sullivan et al., 2015; Shamma et al., 2011; Teki et al., 2013; Tóth et al., 2016). In these figure-ground segregation stimuli, the figure consists of a set of concurrently delivered tones that repeat the *same frequencies* across consecutive time frames, creating a temporally coherent pattern. In contrast, the background tones vary randomly in frequency from frame to frame (see Figure 1). The perceptual salience of the figure increases with the number of repeated (coherent) frequency components and the duration of the cohesive segment, supporting the notion that successful segregation relies on the integration of acoustic information over time and frequency. As in many natural listening environments (e.g., understanding speech in background noise), the figure cannot be detected based on spectral (concurrent) cues alone—it emerges only through temporally integrating information from successive sounds (sequential information) to detect the coherent spectral structure over time.

The temporal coherence theory of auditory scene analysis suggests that stream formation relies on temporal coherence (coincidence operations) between neuronal responses encoding different acoustic features, leading to the binding of coincident responses into perceptual sources (Shamma et al., 2011; Teki et al., 2013). In this view, stream formation begins with cochlear frequency analysis and the extraction of spectral and temporal features, including pitch and location (Lu et al., 2017). Results demonstrate that attentive listening induces rapid modulation of the interactions among neurons, which are driven by the temporal synchronicity of the auditory stimulation, as was shown by changes in the receptive fields of ferrets, such as their adaptation, their response rate, and spiking correlations in primary auditory cortex (Lu et al., 2017; Elhilali et al., 2009). Computational studies have successfully simulated auditory stream formation across various stimuli (Krishnan et al., 2014; Viswanathan et al., 2022). The results align with the assumed mechanisms of temporal coherence analysis, demonstrating that temporal coherence influences speech comprehension in noise; specifically, temporally coherent auditory masker elements interfere with target speech perception (Viswanathan et al., 2022).

### 1.2 Neural correlates of stream segregation by temporal coherence in the human brain

Functional magnetic resonance imaging (fMRI) and electroencephalography (EEG) studies have identified brain regions involved in processing figure-ground segregation in the SFG paradigm, including the auditory cortex and higher-order cognitive areas (superior temporal gyrus, planum temporale, and the intraparietal areas; Boncz et al., 2024; Molloy et al., 2019; O'Sullivan et al., 2015; Shamma et al., 2011; Teki et al., 2013; Tóth et al., 2016). In this paradigm, correct figure detection (separately perceiving the repeating set of tones within the tone cloud) elicits a centrally maximally negative event-related response between 200 and 300 ms from the onset of the tone sequence (Boncz et al., 2024; Tóth et al., 2016). This neural response (object-related negativity; ORN; Alain et al., 2001) has been initially observed for sound segregation based on concurrent (spectral) cues alone, such as when mistuning a partial in a harmonic tone complex, which results in hearing this partial as a separate sound from the rest of the harmonic complex. In the SFG paradigm, the amplitude of the ORN has been shown to increase with both the coherence and the duration of the figure (Tóth et al., 2016), reflecting the increasing salience of a coherent auditory stream. When the figure is task-relevant—e.g., when participants judge the number of perceived sound sources—the ORN is followed by a P400 component (Alain et al., 2001, 2002). These two ERP components are assumed to reflect distinct stages in the formation of auditory object representations: the ORN indexes the brain’s assessment of the likelihood of the presence of a coherent sound source (the figure) over the random background based on the sensory evidence, while the P400 reflects the positive outcome of segregation, i.e., the decision that the target, a separate concurrent sound has been detected (Alain et al., 2001).

### 1.3 Stream segregation abilities in neonates

Initially, research on the development of auditory scene analysis focused on sequential streaming during early infancy using habituation/dishabituation paradigms (see Leibold, 2011, for a comprehensive review). This research revealed that newborns and 3-month-old infants demonstrate sequential streaming, albeit with lower precision than adults (Demany, 1982; McAdams and Bertoncini, 1997; Smith and Trainor, 2011). Consistent with behavioral findings, subsequent research examined neural correlates of sequential stream segregation in infants. These studies suggested that infants also exhibit mismatch negativity (MMN) in response to a version of the “two-streams” paradigm (van Noorden, 1975): two interleaved sound sequences from different frequency ranges with infrequent deviant tones embedded within one or both of the frequency ranges. MMN elicitation in this paradigm by newborn infants suggested that the neural mechanisms for sequential stream segregation are operational already at birth (Winkler et al., 2003). Furthermore, 7-month-olds display an MMN when the deviant occurs within a chord component presented sequentially (Marie and Trainor, 2013).

Recent findings further support newborns’ ability to quickly form representations of auditory regularities, which is critical for processing auditory sequences. After minimal exposure, Tóth et al. (2023) demonstrated that the neonatal auditory system can detect repeating patterns embedded within random tone sequences. Neonates exhibited differential neural responses to regular versus random tone sequences after a single repetition of the pattern. This suggests an innate capacity to rapidly detect sequential auditory regularities, paving the way for future learning and environmental adaptation.

However, whether newborns can segregate streams separated by concurrent cues has not yet received similar effort. Only two studies explored the neural correlates of concurrent stream segregation within the first year of life, and they yielded conflicting results. Both studies employed similar paradigms featuring 500 ms complex tones with mistuned harmonics; half of the tones contained an 8% mistuned second harmonic, while the other half was entirely in tune. While newborns (Bendixen et al., 2015) and 4- to 12-month-old infants (Folland et al., 2015) displayed an ORN in response to mistuned complex tones, 2-month-olds did not (Folland et al., 2015). However, these studies tested only isolated complex tones, thereby precluding the integration of evidence over extended periods. Therefore, in the present study, we tested the functionality of concurrent (spectral) and sequential (temporal) integration together in neonatal auditory stream segregation by presenting temporally coherent tonal figures within a random tone cloud (Teki et al., 2013; see Figure 1).

### 1.4 Research questions and hypotheses

In the present study, high-density EEG was recorded while sleeping newborns were exposed to temporally coherent tonal elements embedded in randomly varying background tones. To investigate the stream segregation processes under different signal-to-noise ratios (SNR) we employed the stochastic figure–ground (SFG) stimulus paradigm, adapted from previous adult and infant studies (Teki et al., 2011, 2013, 2016; Tóth et al., 2016; see Figure 1). Here, the SNR indicates the number of coherent, temporally repeating signal components embedded in a background of randomly varying tones. Each stimulus consisted of a sequence of 30 chords (50 ms per chord). In Signal trials, a subset of tones with fixed frequencies repeated coherently across successive chords for 800 ms, forming a perceptual “figure,” while the remaining tones varied randomly in frequency and constituted the background. Noise-only trials contained only randomly varying tones. To probe stream segregation across different levels of signal salience, Signal trials varied in the number of coherent tones (10, 15, or 20), with the number of background tones decreasing accordingly, yielding Low-SNR, High-SNR, and Signal-only conditions.

The primary research question was whether temporal coherence supports auditory stream segregation already at birth, as reflected in newborns’ event-related brain responses. Based on the temporal coherence theory of auditory scene analysis and prior adult EEG findings using the SFG paradigm, we formulated component-specific hypotheses targeting the object-related negativity (ORN) and the later positive ERP component often referred to as P400 in the adult literature.

First, we hypothesized that signal-containing trials would elicit an ORN-like response relative to Noise-only trials, reflecting the neural detection of a coherent auditory object embedded in a random background. In adults, the ORN indexes the brain’s early, largely automatic assessment of the presence of a distinct sound source (Alain et al., 2001; Tóth et al., 2016). We therefore predicted that, even in newborns, temporally coherent figures would elicit a more negative response than Noise-only trials, indicating an early, automatic form of stream segregation driven by temporal coherence.

Second, we tested whether differences in Signal conditions with varying signal-to-noise ratios would elicit sensitivity levels similar to those of adults. Although adult listeners show graded ORN amplitude modulation as a function of figure salience and coherence strength, we expected that newborns would show limited differentiation between Low-SNR and High-SNR conditions. This prediction reflects the ongoing postnatal maturation of functional networks that integrate acoustic information over time and frequency, which may constrain fine-grained sensitivity to differences in signal strength at birth.

Third, we explored whether a later positive ERP component, potentially corresponding to the P400 observed in awake adults, would be present in newborns. In adults, the P400 has been associated with a later stage of auditory object formation and perceptual evaluation, particularly when the figure is task-relevant (Alain et al., 2001, 2002). Although newborns cannot perform explicit perceptual judgments, we hypothesized that a delayed and possibly attenuated late positivity might nevertheless emerge following signal-containing trials, reflecting an early neural correlate of successful stream segregation. As newborns were tested during sleep and the paradigm involved passive listening, we did not expect this late response to show the same functional characteristics or topography as the adult P400, but rather to represent a developmental precursor of this processing stage.

EEG recordings were conducted during quiet sleep, a standard practice in neonatal research that provides a stable recording environment with minimal movement and reduced sensory interference. Importantly, previous studies have demonstrated that early sensory and perceptual ERP components in newborns—such as the mismatch negativity and other sound-related responses—are robustly elicited during sleep and show functional similarities to responses observed during wakefulness, albeit with longer latencies and developmental differences in scalp distribution (Winkler et al., 2003; Bendixen et al., 2015; Tóth et al., 2023). Because the present paradigm relied on passive auditory stimulation and automatic segregation mechanisms rather than attention or task engagement, quiet sleep was considered an appropriate and methodologically justified state for probing the neural bases of auditory stream segregation at birth.

## 2 Methods

### 2.1 Participants

Forty-seven healthy full-term newborn infants (0–4 days of age, APGAR score 9/10 or above) were measured. They had a mean gestational age of 280.35 days (40 + 3 weeks; SD = 8.12 days) and a mean birth weight of 3,549 g (SD = 434.67 g). All newborns included in the study passed the routine universal newborn hearing screening conducted at the maternity ward (based on otoacoustic emissions and/or automated auditory brainstem responses, according to hospital protocol). Only infants with documented normal screening results and without known risk factors for hearing impairment were included in the study. Issues due to technical failures in the data-acquisition machinery (the stimulus markers were not recorded) led to the exclusion of 5 newborns, while preprocessing steps (see section 2.5. EEG preprocessing) led to the exclusion of another nine newborns. Thus, the final sample included 33 infants (15 males, 18 females). Informed consent was obtained from one or both parents, and the infant’s mother could opt to be present during the recording. The study fully complied with the World Medical Association of Helsinki and all national and international laws. The Scientific and Research Ethics Committee of the Medical Research Council of Hungary (ETT TUKEB) granted permission for the research.

### 2.2 Stimuli

Stochastic figure–ground (SFG) stimuli were adapted from previous studies (Teki et al., 2011; Tóth et al., 2016). SFG stimuli consisted of a background of randomly varying frequency pure tones (noise) and, in some conditions, an additional set of pure tones with fixed frequencies repeating across successive time windows, forming a temporally coherent figure (signal).

The primary experimental manipulation was the number of temporally coherent frequency components forming the figure. For convenience, we refer to this manipulation as the *signal-to-noise ratio (SNR)*, defined here as the number of coherent (signal) components embedded in a background of randomly varying tones. Although coherence strength in SFG paradigms is more commonly described in terms of the number of repeating elements, the term SNR is used in the present study as a shorthand to denote the relative proportion of coherent components, rather than acoustic energy in the classical sense.

Four stimulus conditions were used: Noise-only, Low-SNR, High-SNR, and Signal-only (200 stimuli per condition). In the Noise-only condition, stimuli contained no coherent components (0 signal components). In the Low-SNR and High-SNR conditions, stimuli contained 5 and 10 coherent frequency components, respectively. In the Signal-only condition, all 20 components were coherent. The four stimulus conditions are illustrated in Figure 1A in time–frequency space.

Each stimulus had a total duration of 1,500 ms and consisted of 30 consecutive time windows, each 50 ms in duration. Each time window contained 20 pure tones of different frequencies, resulting in a 30 × 20 time–frequency grid (Figure 1A). In stimuli containing a signal (all except Noise-only), an 800-ms window was pseudo-randomly selected to include the coherent components, with the constraint that the first 300 ms of each stimulus contained only noise. This randomization ensured the figure’s onset was unpredictable.

In the Noise-only condition, all 20 frequency components varied randomly from time window to time window throughout the entire stimulus. In the Signal conditions, the selected 800-ms window contained 5, 10, or 20 fixed frequency components (Low-SNR, High-SNR, and Signal-only, respectively). In contrast, the remaining components (15, 10, or 0, respectively) varied randomly in frequency, as in the Noise-only condition. Thus, the signal differed from the noise solely in its temporal structure, namely the repetition of fixed frequencies across successive time windows, and could not be distinguished based on intensity or spectral cues alone. Each 50-ms time window contained 20 pure tones (starting at zero phase), with a 10-ms raised-cosine ramp applied at both the onset and the offset. The frequencies were randomly selected from a 64-step set, logarithmically spaced between 500 and 4,000 Hz. low-frequency sound transmission in newborns is strongly affected by immature middle-ear mechanics and residual middle-ear fluid, leading to increased attenuation and variability below approximately 400–500 Hz (Keefe et al., 1993; Keefe and Levi, 1996).

Following the protocol of Teki et al. (2011), all pure-tone components had equal amplitudes, without controlling for perceived loudness. Because the assignment of frequency components to signal or noise was randomized across stimuli, perceived loudness and saliency could not serve as reliable cues for distinguishing the figure from the background. Stimuli were presented at a comfortable overall intensity of approximately 70 dB SPL.

All stimuli were generated with Matlab (R2017a, Mathworks; Natick, MA, USA) at a sampling rate of 44.1 kHz and 16-bit resolution and delivered using a Maya 22 USB external sound card and ER•2 Insert Earphones (Etymotic Research Inc., Elk Grove Village, IL, USA), inserted into the infants’ ears using ER2 Foam Infant Ear-tips.

### 2.3 Procedure

EEG recordings were conducted in a dedicated experimental room at the Department of Obstetrics and Gynecology, Szent Imre Hospital, Budapest. The newborn participants were asleep during stimulus presentation, with sleep states classified according to the criteria established by Anders and Weinstein (1972). Only infants who remained in quiet sleep for the entire 45-min duration were included in the study. In addition to behavioral criteria, the EEG signal was visually examined to ensure muscle tension was stable, respiration was regular, and significant eye movements were absent.

A total of 800 trials (200 for each of the four conditions: Noise-only, Low-SNR, High-SNR, Signal-only), were presented. Stimuli were delivered within a single experimental block, with the order of the conditions randomized. Stimulus presentation was controlled using Psychophysics Toolbox (Kleiner et al., 2007) (Psychology Software Tools, Inc., Pittsburgh, PA, USA), and EEG activity was recorded continuously throughout the presentation. The silent inter-stimulus interval (ISI, offset to onset) duration was randomized between 800 and 1,200 ms with values rounded to 100. The experimental session lasted approximately 45 min.

### 2.4 EEG data acquisition

ActiChamp Plus amplifier with a 64-channel sponge-based electrode system (R-Net, saltwater sponges and passive Ag/AgCl electrodes) and BrainVision Recorder were employed for EEG recording (all from Brain Products GmbH, Gilching, Germany). The sampling rate was 500 Hz with a 100 Hz online low-pass filter applied for visualization-only purposes. Electrodes were placed according to the International 10/20 system. The Cz electrode served as the reference electrode, while the ground electrode was placed at the center of the forehead. During the recording, impedances were kept below 15 kΩ.

### 2.5 EEG preprocessing

EEG data were imported to MATLAB (Mathworks, Natick, MA, USA; ver. 2021a) and preprocessed in EEGLab (Delorme and Makeig, 2004) using the validated NEAR pipeline (Kumaravel et al., 2022). In the NEAR pipeline, data were first band-pass filtered between 0.1 and 100 Hz with a finite impulse response (FIR) filter. Cut-off frequencies were 0.05 and 100.05 Hz (−6 dB). Then, the Local Outlier Factor (LOF), a data-driven approach based on the squared Euclidean distance across electrodes, was applied to remove noisy channels (Kumaravel et al., 2022). Following the recommendations of Kumaravel et al. (2022) for newborns, we set the threshold at 2.5.

This was followed by Artifact Subspace Reconstruction (ASR; Mullen et al., 2015): channels were deemed ‘bad’ and removed if either flat for longer than 5 s or through computation of each channel’s correlation to its RANdom SAmple Consensus (RANSAC) reconstruction for each window. The correlation threshold for channel removal was set at 0.8. The maximum number of channels rejected was 11. A Principal Component Analysis (PCA) decomposition was then applied to all recordings to identify artifactual PCs (defined by comparison with the cleanest parts of the data) and reject them, and to reconstruct activity from the remaining components. The windows’ standard Deviation (SD) was set at 20. After all ASR steps, the overall mean number of electrodes rejected was 6.51 (SD = 2.31). The rejection threshold was set to 11: participants with more than 11 noisy electrodes were excluded from further analysis. This threshold was chosen based on reasonable ranges in developmental samples. This led to the exclusion of 9 participants, as noted in Section 2.1. Participants. The removed electrodes were spherically interpolated using the full-rank electrode matrix, and the data were re-referenced to the average reference.

The trials were then epoched into −200 to 1,000 ms segments relative to the beginning of the signal in the signal conditions. In the noise-only condition, time points for triggering were selected pseudo-randomly by using the onset value of a trigger in one of the signal trials. The −200 to 1,000 ms range was chosen because target responses reportedly arise within the first 500 ms in newborns (Bendixen et al., 2015), and to minimize the number of epochs rejected due to late artifacts (beyond our focus of interest). After baseline correction, epochs containing abrupt amplitude changes were rejected using a threshold of ±75 μV. At the same time, a joint probability-based rejection was used to remove epochs exceeding 3 SD from the mean of activity across channels. The average number of remaining epochs was 135.44 (SD = 20.17) for the Noise-only condition, 136.29 (SD = 18.96) for the Low-SNR condition, 134.11 (SD = 20.6) for the High-SNR condition, 137.62 (SD = 16.16) for the Signal-only condition (543.48, SD = 72.98 for the total number of epochs per infant). The proportion of rejected epochs is similar to that in other studies of neonates, and rejections are typically attributed to neonates moving during sleep, poor electrode adherence to the scalp, and occasional high-amplitude electromagnetic signals in the hospital environment. A series of t-tests was performed to assess differences in epoch numbers across the four conditions, but none were found (lowest *p* = 0.44).

### 2.6 ERP analysis

**Figure 2:**
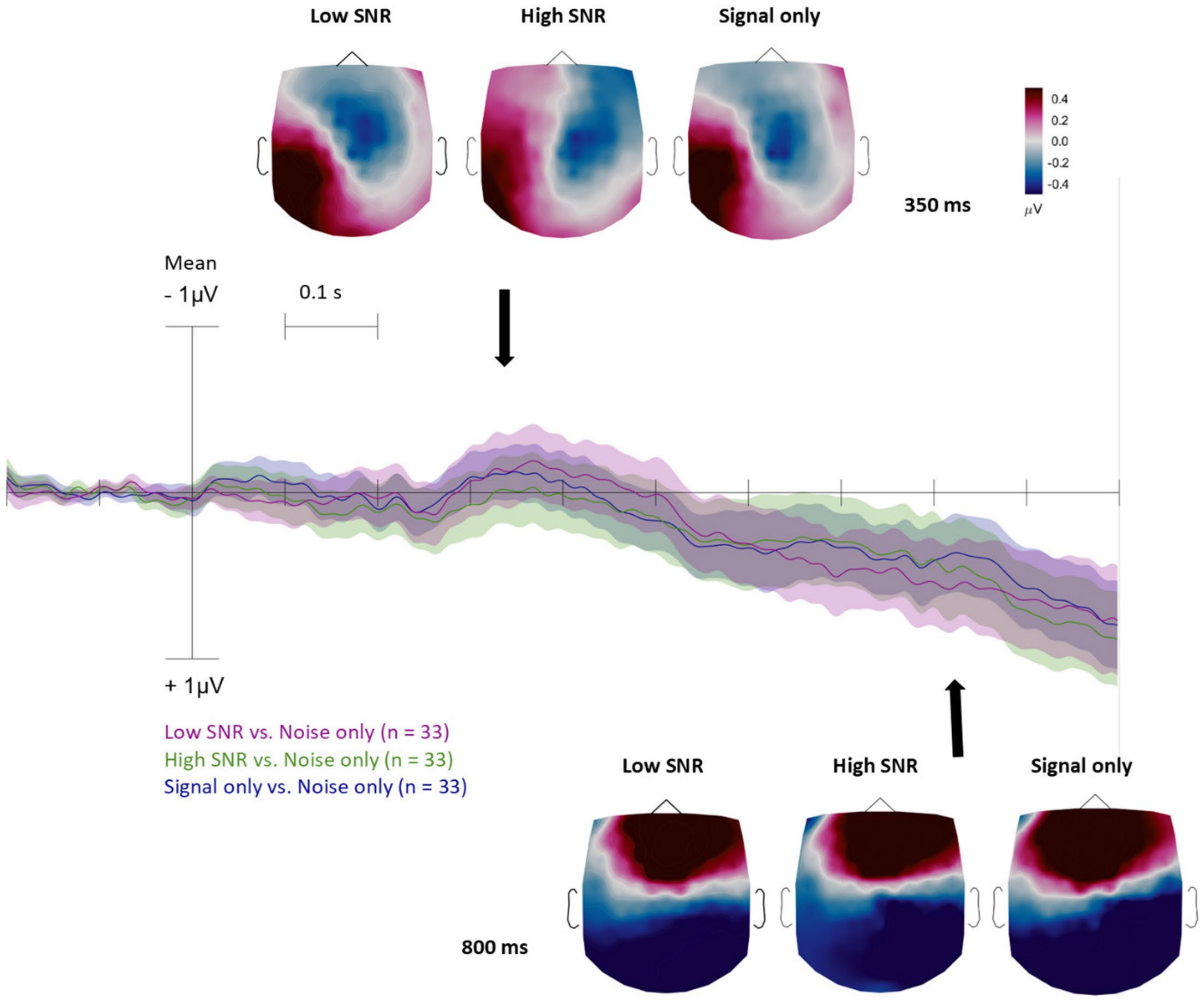
Group-average (*N* = 33) scalp distribution and ERP waveforms for the difference between the Signal conditions (Low-SNR, High-SNR, and Signal-only) and the Noise-only condition. Topoplots highlight the scalp distribution of the difference waveforms at the peak of the early response (top of the figure) and a characteristic latency of the late slow shift (bottom right of the figure)–see the color scale for the difference amplitudes. The difference waveforms are shown for the means of channels Fp1, Fz, F3, F7, FC5, FC1, FC2, F4, Fp2, AF7, AF3, AFz, F1, F5, FT7, FC3, FC4, FT8, F6, F2, AF4 with color marking the condition. Confidence areas represent standard errors.

Before ERP analysis, the EEG data were low-pass filtered at 45 Hz. For each signal-containing condition (Low-SNR, High-SNR, and Signal-only), we computed difference waveforms by subtracting from the response the ERP obtained in the Noise-only condition. Visual inspection of the difference waveforms revealed two distinct components with different scalp distributions. An early component, occurring between 300 and 600 ms, exhibited a temporo-parietal maximum, whereas a late component, occurring between 600 and 1,000 ms, showed a predominantly fronto-central distribution (Figure 2).

Based on these observed spatial and temporal characteristics, we restricted further statistical testing to these two time windows and to electrode groups corresponding to the visually identified topographies, frontal and temporo-parietal electrodes for the early window (Fz, F3, FC2, AFz, F1, F2, F7, FC5, FC3, F5, FT7, C3, C5, CP3, CP5, P7, P9, P5, T7, and TP7), and fronto-central electrodes for the late window (Fp1, Fz, F3, F7, FC5, FC1, C3, FC2, FC6, F10, F8, F4, Fp2, AF7, AF3, AFz, F1, F5, FT7, FC3, FC4, FT8, F6, F2, AF4, AF8, T7, CP5, P7, P9, T8, C1, C5, TP7, CP3, P5, C6, and C2). To ensure that the results were not driven by gender differences, we conducted independent-samples t-tests on the averaged values across the two time windows by gender. No significant effects were observed (all *p*s > 0.29). As an exploratory analysis, we examined correlations between maturational indices, defined as *z*-scores of maternal age, gestational age at birth, birth weight, birth length, and head circumference, and the mean values obtained from the early and late time windows.

Then, permutation-based one-tailed t-tests were used to assess (1) whether absolute voltage values at individual time points were significantly higher than those observed in the Noise-only condition, analyzed separately for each signal condition, using one-tailed tests because our comparisons were made against the Noise-only condition, which represented our theoretical zero value and for which our hypotheses predicted a specific direction of effect: signal-containing conditions were expected to produce greater responses than Noise-only, and (2) whether the signal conditions significantly differed from one another, for which two-tailed tests were used. Permutation testing is a non-parametric statistical approach that builds the “null” distribution by repeatedly shuffling condition labels. In our analysis, condition labels were randomly permuted across subjects, and the test statistic (e.g., *t*-value) was recalculated for each permutation. Repeating this procedure 1,000 times yielded an empirical null distribution, against which the observed statistic was compared to obtain a permutation-based *p*-value (*p* < 0.05). This method offers two main advantages: it avoids assumptions about the distribution of the data and multiple-comparison issues. Statistical analyses were conducted using the Brainstorm toolbox (Tadel et al., 2011) in Matlab (ver. 2024a).

### 2.7 Transparency and openness

Data are available on request due to privacy and ethical restrictions.

## 3 Results

Brain activity significantly differed from the Noise-only condition in all three signal conditions (Low-SNR, High-SNR, and Signal-only). Moreover, the Low-SNR condition was significantly different from the Signal-only condition. No other significant differences were observed between the signal conditions (Low- vs. High-SNR; High-SNR vs. Signal-only).

**Figure 3:**
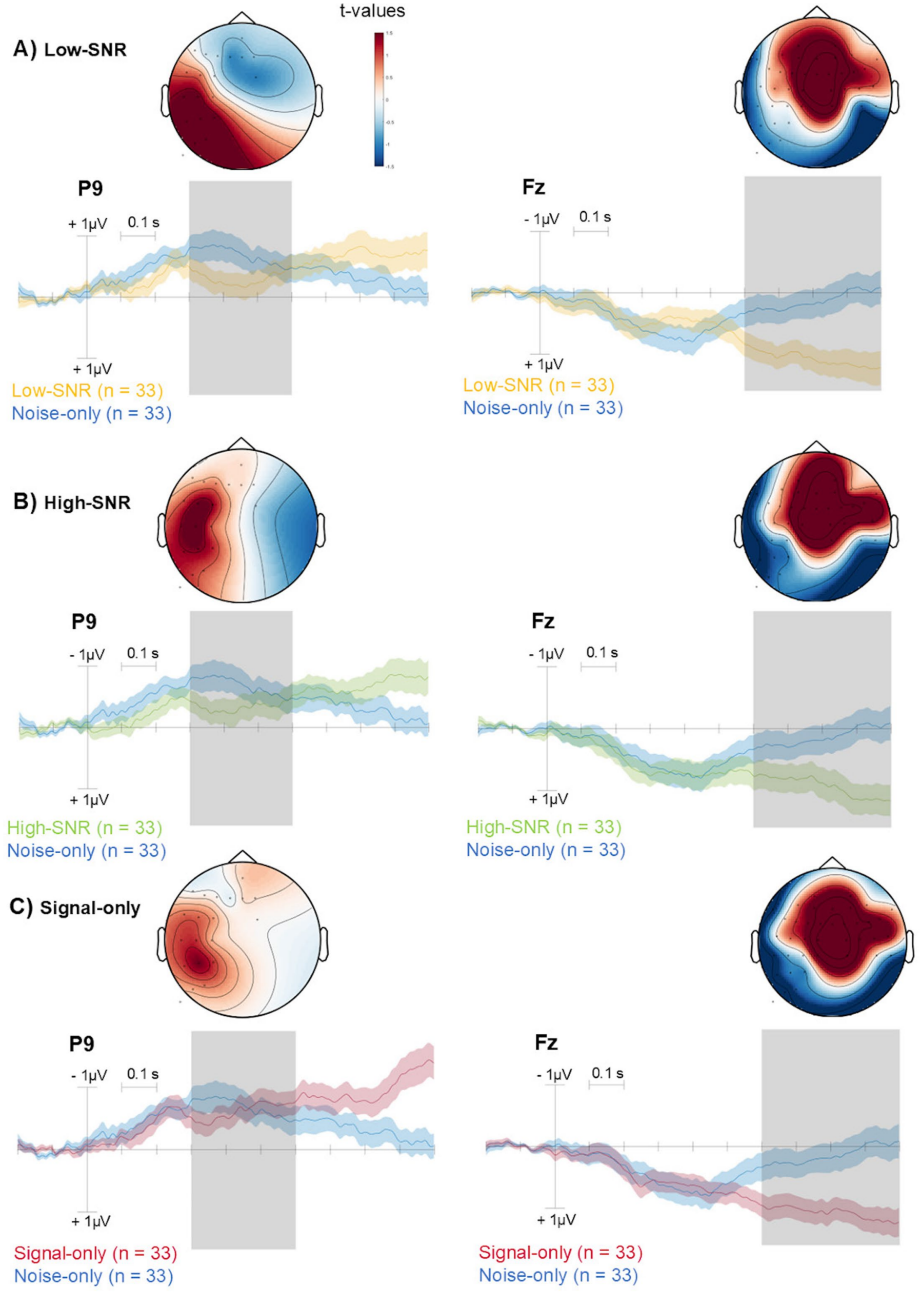
Topology (scalp distribution) of *t*-values from permutation testing is shown for the early (300–600 ms) and late (600–1000 ms) time windows (indicated in gray), highlighting differences between Signal conditions and the Noise-only condition **(A-C)**. *T*-values are represented in color [see color scales in **(A)**]. The ERP waveforms are shown for selected channels (P9, Fz) that exhibited maximal differences across permutation testing for illustrative purposes. Confidence areas represent standard errors.

More in depth, permutation-based t-tests confirmed that amplitudes during the early (300–600 ms) and late (600–1,000 ms) windows were significantly greater than zero across both temporo-parietal (early window) and fronto-central (late window) electrodes for all three signal conditions (*p* < 0.05; Figure 3). This pattern reflects progression in time in the neural response, from posterior to anterior scalp regions.

**Figure 4:**
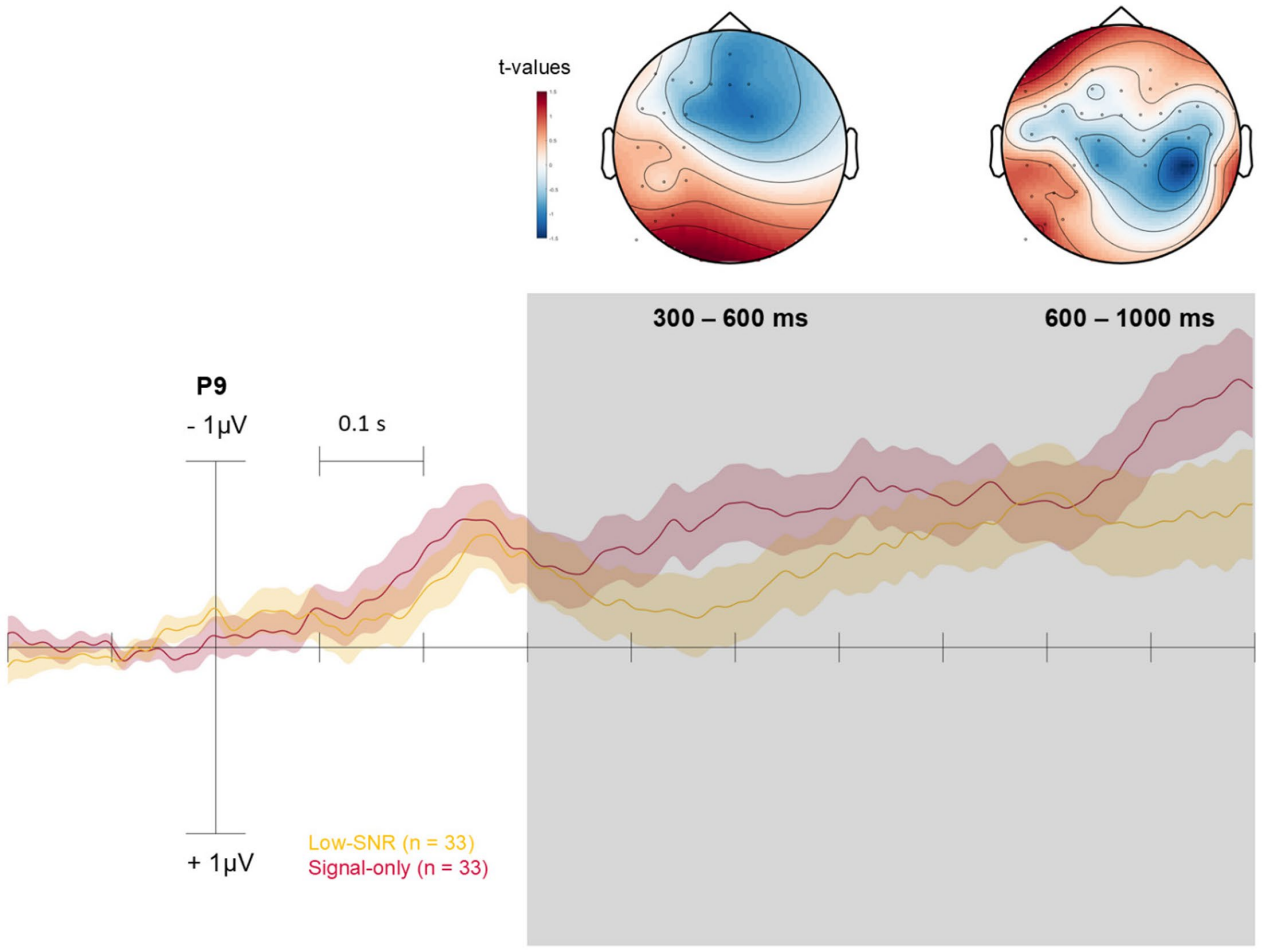
Topology (scalp distribution) of *t*-values from permutation testing is shown for the early (300–600 ms) and late (600–1000 ms) time windows (indicated in gray), highlighting differences between the Low-SNR and the Signal-only condition. *T*-values are represented in color (see color scales). The ERP waveforms are shown for P9 for illustrative purposes. Confidence areas represent standard errors.

Regarding the between-condition comparisons, we observed a significant difference between the Low-SNR and the Signal-only conditions, with increased parietal activity in the Low-SNR condition during both the early and the late time windows (*p* < 0.05; Figure 4). In contrast to comparisons between the signal conditions and the Noise-only condition, the activity in the Low-SNR vs. Signal-only comparison remained focused over parietal areas across both windows. No other significant differences were observed between the remaining condition pairs.

Although correlations with maturational indices were not statistically significant (all *p*s > 0.9), some patterns may warrant future investigation. Specifically, we observed a negative correlation between the early window and gestational age at birth (*r* = −0.17), a positive correlation between the late window and birth weight (*r* = 0.22), and a positive correlation between the early window and head circumference at birth (*r* = 0.26). Graphical representation of these relationships can be found in the Supplementary material.

## 4 Discussion

The results provide evidence for the role of temporal coherence in organizing auditory scenes from birth. This study demonstrates that newborns can segregate auditory streams based on temporal coherence, indicating that the ability to segment sensory input into discrete objects (Winkler and Denham, 2024) is already present at birth. By examining difference waveforms and their spatial distributions, we identified two temporally distinct ERP responses: an early response between approximately 300–600 ms, with a temporo-parietal maximum, and a later response between 600 and 1,000 ms, characterized by a more fronto-central distribution. Both responses were observed for signal-containing conditions relative to the Noise-only condition.

By calculating difference-waveforms and examining their spatial distributions, we identified two distinct ERP components: an early response between 300 and 600 ms with a temporo-parietal maximum, and a later response between 600 and 1,000 ms with a fronto-central distribution. These components were observed across all three signal conditions (Low-SNR, High-SNR, and Signal-only), relative to the Noise-only condition.

In adult listeners, auditory figure–ground segregation is typically associated with the object-related negativity (ORN), followed by a later positive component such as the P400 (Alain et al., 2001; Johnson et al., 2007). In the present newborn sample, however, the early response differed from the canonical adult ORN in several respects, including its polarity, scalp distribution, and hemispheric lateralization. We therefore interpret this early activity not as a direct homolog of the adult ORN, but rather as an ORN-like or putative object-related response, potentially reflecting an early developmental stage of auditory object processing. Such deviations from adult ERP morphology are expected in neonates. Early auditory responses in infancy often differ in polarity, latency, and topography due to immature cortical layering, incomplete myelination, and developing long-range connectivity (Adibpour et al., 2020; Long et al., 2018; Polver et al., 2023). From this perspective, the early response observed here may represent a developmental precursor of later object-related components that become more focal, midline-centered, and negative in polarity over the course of maturation.

Visual inspection of the scalp distributions suggests that the ORN-like response in newborns may be relatively stronger over the right hemisphere. In adults, figure–ground segregation and temporal coherence–based auditory object formation have been shown to preferentially engage right auditory cortical regions, including the planum temporale and superior temporal gyrus, particularly for non-speech and spectrally complex stimuli (Teki et al., 2013; Molloy et al., 2019). Developmental neuroimaging studies indicate that hemispheric asymmetries in auditory processing are already detectable in early infancy, although they remain less specialized and more variable than in adults (Dehaene-Lambertz and Pena, 2001; Minagawa-Kawai et al., 2011). In newborn EEG, apparent lateralization effects may further be shaped by ongoing cortical maturation, skull conductivity, and individual anatomical variability. Consequently, while the observed right-leaning asymmetry may reflect an early bias in the neural mechanisms supporting auditory scene analysis, it should be interpreted cautiously. Longitudinal and source-level studies will be necessary to determine whether such asymmetries are stable across development and whether they relate to later specialization for speech, music, or other complex auditory functions.

Although newborns were asleep during testing—precluding a definitive identification of the P400—the fronto-central activity observed in the 600–1,000 ms window may reflect a late positivity that could be a precursor to the P400. This later fronto-central positivity was more robust and aligns more closely with the P400 described in adults. Although newborns were tested during quiet sleep—precluding explicit perceptual reports—the presence of this late positivity suggests that higher-level evaluation of temporally coherent auditory input can occur even in the absence of attention or conscious awareness. This interpretation is consistent with evidence that newborns can learn and form auditory representations during sleep (Fifer et al., 2010). Its presence during passive sleep suggests that some aspects of auditory scene evaluation can occur without conscious awareness. Newborns have been shown to learn during sleep (Cheour et al., 2002; Fifer et al., 2010). Our findings partially diverge from previous work on auditory novelty detection, which reported responses in preterm infants only during active sleep, whereas basic sensory perception and responses to standard tones were observed in both active and quiet sleep (Suppiej et al., 2010; Kushnerenko et al., 2013).

In contrast, our results indicate that even during quiet sleep, newborns are capable of processing auditory scenes, underscoring their functional relevance. Both the early negative wave—emerging around 300 ms over central and temporo-parietal regions—and the subsequent fronto-central positivity resemble adult ERP components (Boncz et al., 2024; Tóth et al., 2016), suggesting continuity in the developmental trajectory of auditory processing. However, the longer latencies observed in newborns compared to adults are consistent with maturational delays in neonatal auditory processing, likely reflecting ongoing myelination and synaptic development in primary and secondary auditory areas, which support processes critical for temporal binding and auditory stream segregation (Adibpour et al., 2020; Long et al., 2018). Indeed, infants have been reported to exhibit longer response latencies earlier in development, with these differences gradually decreasing as they mature (Polver et al., 2023). Future studies should include a range of age groups to better map the developmental trajectory of response latencies. Although our exploratory correlations between maturational indices (e.g., birth weight, gestational age, head circumference) and early and late response latencies were not significant, the observed trends suggest that early growth characteristics may still be relevant for understanding developmental changes in response timing. Future studies including multiple age groups will be essential for mapping how figure salience influences response latency across developmental stages.

Taken together, our findings indicate that while the late P400-like response provides the clearest evidence for auditory stream segregation at birth, the earlier ORN-like activity may reflect an immature precursor of object-related processing. Future longitudinal work will be essential to determine how these early and late responses evolve into the canonical adult ERP components associated with auditory scene analysis.

Consistent with this interpretation, the Low-SNR condition elicited significant differences in neural responses over parietal areas compared to the Signal-only condition. In contrast to adult listeners, newborns did not show a monotonic increase in neural response amplitude with increasing figure coherence. Instead, the only reliable difference between signal conditions was observed between the Low-SNR and Signal-only conditions, with increased parietal activity in the Low-SNR condition. Importantly, this pattern differs from adult findings, in which both ORN and P400 amplitudes scale positively with figure coherence and duration (Tóth et al., 2016). We therefore interpret the Low-SNR effect not as evidence for enhanced segregation at lower coherence levels, but rather as reflecting increased processing demands when temporally coherent elements are embedded in a more complex and acoustically variable background. In newborns, immature temporal integration mechanisms may lead to greater neural engagement under more challenging listening conditions, without yielding stronger or more stable perceptual object representations. In contrast, the Signal-only condition may place lower demands on segregation mechanisms, resulting in reduced engagement of distributed parietal networks. The absence of systematic differences between the Low- and High-SNR conditions further suggests that newborn auditory processing does not yet exhibit the graded sensitivity to figure coherence observed in adults. This non-monotonic pattern likely reflects developmental immaturity in temporal coherence processing and underscores the fact that adult-like scaling of auditory object representations emerges only later in development.

The use of permutation-based statistical testing enabled us to identify robust effects in both the early and late time windows without relying on assumptions of normality—an important consideration given the variability of neonatal EEG data. These tests confirmed that the topography of neural activity shifted significantly over time: from a temporo-parietal distribution in the early window to a more fronto-central pattern in the later window. This dynamic spatial progression may indicate a transition from initial sensory/perceptual processing to higher-level evaluation of the auditory input. Similar topographic shifts have been observed in adults during auditory figure-ground segregation, which are thought to reflect increased interaction between primary auditory cortices and higher-order associative areas (Diepenbrock et al., 2017; Mishra et al., 2021; Viswanathan et al., 2022).

These findings contribute to a growing body of evidence suggesting that the basic neural architecture supporting auditory stream segregation is present from birth. The temporally and spatially distinct ERP components observed in our study reflect an emerging capacity for detecting salient auditory events in complex soundscapes. This capacity likely serves as a foundation for the development of selective attention and speech perception (Elhilali et al., 2009; Kujala et al., 2023; Rezaeizadeh and Shamma, 2021; Stefanics et al., 2007), enabling infants to orient toward relevant signals such as the caregiver’s voice (Winkler et al., 2003; Kujala et al., 2023) and to extract wordforms from auditory streams (Kujala et al., 2023).

Future work employing source reconstruction and connectivity analysis could further clarify the cortical areas generating these early and late components, as well as their developmental trajectories. Longitudinal studies could also examine how the maturation of spatially distinct ERP components relates to the emergence of language, attention, and social communication abilities. In summary, this study provides compelling evidence that human newborns can segregate temporally coherent auditory input from background noise. The observed spatiotemporal dynamics—marked by early sensory and later associative responses—highlight the foundational neural mechanisms of auditory scene analysis at birth. These mechanisms likely support early learning through observation and interaction, laying the groundwork for the development of communication and cognition.

## Data Availability

The raw data supporting the conclusions of this article will be made available by the authors without undue reservation.
