# Supplementary material for: Evidence for temporal-coherence-based segregation of complex auditory scenes in the newborn human brain
Source: Front Hum Neurosci. 2026 Apr 1;20:1719515. doi: 10.3389/fnhum.2026.1719515 (PMC13079306; doi:10.3389/fnhum.2026.1719515)
Supplement: Supplementary file 1 [file Data_sheet_1.docx]

**Supplementary**


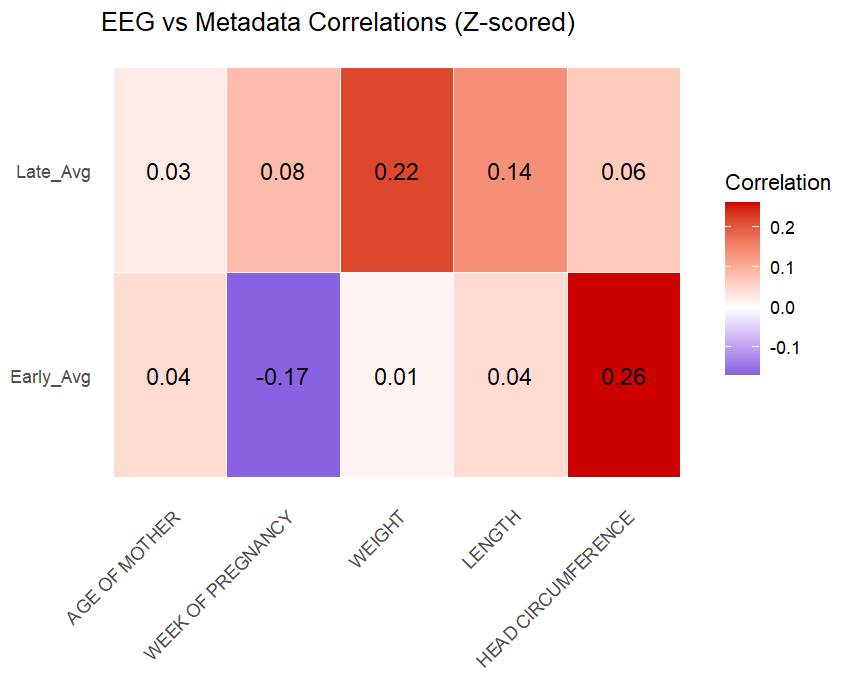


Supplementary Figure. Correlation matrix of maturational indices and averaged values of responses across time windows. This figure displays the z-scored maturational indices—maternal age, gestational age at birth, birth weight, birth length, and head circumference—and their correlations with averaged response latencies in early and late time windows. Although no correlations reached statistical significance (all ps > .9), trends such as a negative association between early-window latency and gestational age, and positive associations between early-window latency and head circumference and between late-window latency and birth weight, are visible.
